# The Incorporation of Low-Molecular Weight Poly(Mannitol Sebacate)s on PLA Electrospun Fibers: Effects on the Mechanical Properties and Surface Chemistry

**DOI:** 10.3390/polym14163342

**Published:** 2022-08-16

**Authors:** Víctor Hevilla, Águeda Sonseca, Enrique Gimenez, Coro Echeverría, Alexandra Muñoz-Bonilla, Marta Fernández-García

**Affiliations:** 1 Instituto de Ciencia y Tecnología de Polímeros (ICTP-CSIC), C/Juan de la Cierva 3, 28006 Madrid, Spain; 2 Interdisciplinary Platform for “Sustainable Plastics towards a Circular Economy” (SUSPLAST-CSIC), 28006 Madrid, Spain; 3 Instituto de Tecnología de Materiales, Universitat Politècnica de València, Camino de Vera, s/n, 46022 Valencia, Spain

**Keywords:** mannitol, PLA, fibers, alignment, mechanical properties, modification

## Abstract

We offer a report on the synthesis of low-molecular weight biobased poly(mannitol sebacate) (PMS) and its functionalization with acrylate groups (PMSAc). These synthesized polyesters were blended at a low level (10 wt%) with poly (lactic acid) PLA to prepare aligned fibers by electrospinning, coupled with a rotatory collector. The obtained fibers were extensively studied by Fourier transform infrared spectroscopy (FTIR), scanning electron microscopy (SEM), differential scanning calorimetry (DSC), and wide-angle X-ray diffraction (WAXS), employing synchrotron radiation. The incorporation of the PMSs on the PLA fibers did not significantly affect the fiber diameters, whereas the alignment was almost maintained. The crystallinity and thermal properties were also slightly modified with the addition of PMSs, and an increase in the degree of crystallinity and in the glass transition temperature of the blend compared to PLA was observed. Remarkably, the PLA/PMSs fibers were more ductile due to the elastomeric character of PMS, with higher values of elongation at break and tensile strengths, and a smaller Young modulus in comparison with the PLA fibers. These modifications of the properties were more noticeable in the case of the acrylated PMS, which also provided readily available functional groups at the surface for further chemical reactions, such as the Michael addition or crosslinking processes.

## 1. Introduction

Today, the European Green Deal that was approved in 2020 requires the efficient use of resources and protection of nature to avoid contamination and minimize carbon footprints. One of the strategies for addressing this challenge focuses on the research toward the development of more sustainable materials. This implies the use of sustainable raw materials and also the use of more sustainable processing methods that could contribute to minimizing the energy expenditure in their production, as well as the use of low processing temperatures, for example.

Currently, one of the most frequently used processing techniques in polymer science and technology is electrospinning [1]. This is an inexpensive and effective process, which can easily produce continuous polymeric micro- or nanofibers with 3D-topography (scaffolds), high porosity, a large surface area, etc. [2,3,4,5,6]. The mechanism behind the obtainment of the fibers involves the application of an electrostatic force to generate a polymeric jet towards a collector electrode [1]. The obtained fiber properties can be fine-tuned by applying different set-up conditions, different collectors (plates, rotating drums, etc.), and parameters to fulfill the requirements of the desired potential application for the final material. For instance, recent studies demonstrated that the production of aligned electrospun fibers by means of a rotating drum gives rise to fiber mats with potential applications in wound healing. This fiber arrangement mimics the extracellular matrix, in addition to contributing towards improve the cell viability compared to random fibers. For all these reasons, electrospun membranes are ideal materials for applications in different areas, such as energy storage (supercapacitors) [7], electronic applications (membranes for Li-ion batteries) [7], and in biomedicine [8,9] with a particular applicability to tissue engineering [10,11,12].

For the development of sustainable materials, biobased and natural polymers or biopolymers have attracted interest in relation to electrospinning processing techniques. They are postulated as plausible candidates to substitute fossil fuel-derived polymers, also possessing properties such as biocompatibility and biodegradability. For instance, poly (lactic acid) (PLA) [13] has been extensively studied for its potential in biomedical uses, among other applications. However, this polymer has drawbacks that limit its use in certain bio-applications, such as tissue engineering or tissue regeneration. For instance, PLA is sensitive to temperature and has a low rate of degradation, a poor cellular behavior, a moderate inflammatory response, and low toughness. To overcome some of these problems, recent studies have focused on strategies of blending PLA with other materials to improve its mechanical properties and, thus, expand its range of applications [14,15,16,17,18]. For this purpose, polyol polyesters are good candidates. These polymers are made from renewable resources, such as glycerol, sorbitol, mannitol, and diacids, and can also be used for a large number of applications, such as elastomers, sealants, and adhesives, etc. [19,20,21,22] They are generally environmentally degradable and, through prolonged contact with tissues, polyol polyesters are hydrolyzed to their natural building blocks, from which are built up [23,24]. Therefore, they are good candidates for improving the physicochemical characteristics of PLA. Among the polyol polyesters, the family of poly (polyol sebacate)s are probably some of the most appropriate for this purpose. These polymers have a degradation mechanism that provokes a linear loss of mass and mechanical properties throughout the process instead of dramatically diminishing these properties. Therefore, the material typically maintains its geometry, performance, and stiffness, being more compatible with soft tissues [2,3].

Among this family is poly(mannitol sebacate) (PMS), which is made from mannitol, a diastereomer of sorbitol (the most well-studied natural polysaccharide-derived hexitol), and sebacic acid (a natural C10 fatty acid), which is directly produced from castor oil. The main problem affecting its synthesis is the high melting point of the mannitol, as well as its low solubility in organic solvents, the reasons for which it is formed by melt polycondensation [20]. PMS has been used as an upconverting cross-linked elastomer with covalently tethered chromophores [25], but mainly it is used as a matrix in blends and nanocomposites with an increased modulus and enhanced strain and toughness [14,22,26], which are requirements for applications in surgical procedures and tissue engineering [19,20].

In addition to this, PMS has pendant hydroxyl groups in its structure, which can be functionalized with other molecules and, therefore, provide new properties to PMS, such as antimicrobial activity and/or antioxidant activity. Thus, the aim of this work is to obtain aligned fibers from the blending of PLA and PMS and the blending of PLA and acrylated PMS (PMSAc) by means of electrospinning, coupled with a rotatory collector. Our objective is twofold. On one hand, we aim to study the effects of the combination of PMS and PMSAc on the properties of the obtained fibers, and secondly, we aim to demonstrate the potential of the surface functionalization of PLA/PMSAc fibers as a proof of concept, in order to provide additional functionalities to the fiber mats. The resulting fibers are analyzed in terms of their morphology, structural properties and crystallinity, thermal properties and stability, and mechanical properties.

## 2. Experimental

### 2.1. Materials

Sebacic acid (SA, HOOC-(CH_2_)_8_-COOH), mannitol (MA, C_6_H_14_O_6_), triethylamine (TEA, N(CH_3_)_3_), acryloyl chloride, hydrochloric acid (HCl), anhydrous N,N-dimethylformamide (DMF), deuterated dimethyl sulfoxide (DMSO-d6), chloroform (CHCl_3_), methanol (MeOH), lithium bromide (LiBr), and 7-mercapto-4-methylcoumarin (≥97.0%) were acquired from Sigma Aldrich and used as they were received. Biodegradable poly(lactic acid) (PLA) was supplied by NatureWorks^®^ (Plymouth, MN, USA) under the trade name PLA6202D, with <2% of D-lactic acid monomer and a weight-average molecular weight (M_w_) of 157.5 kDa.

### 2.2. Synthesis of the Poly(Mannitol Sebacate) and Posterior Acrylation

The synthesis of the PMS was carried out with sebacic acid and D-mannitol in bulk. SA and MA were reacted in a molar ratio 1/1.1 in a 250 mL three-necked round-bottomed flask. The temperature was slowly increased to 150 °C under continuous stirring and nitrogen flow, and the reaction lasted for 7 h. The resulting polymer was dissolved in DMF (150 mg/mL), filtered and purified by dropwise precipitation into a fourfold excess of cold ultrapure water, under continuous stirring. The precipitate was collected and dried in a vacuum until a constant weight was obtained.

**Scheme 1:**
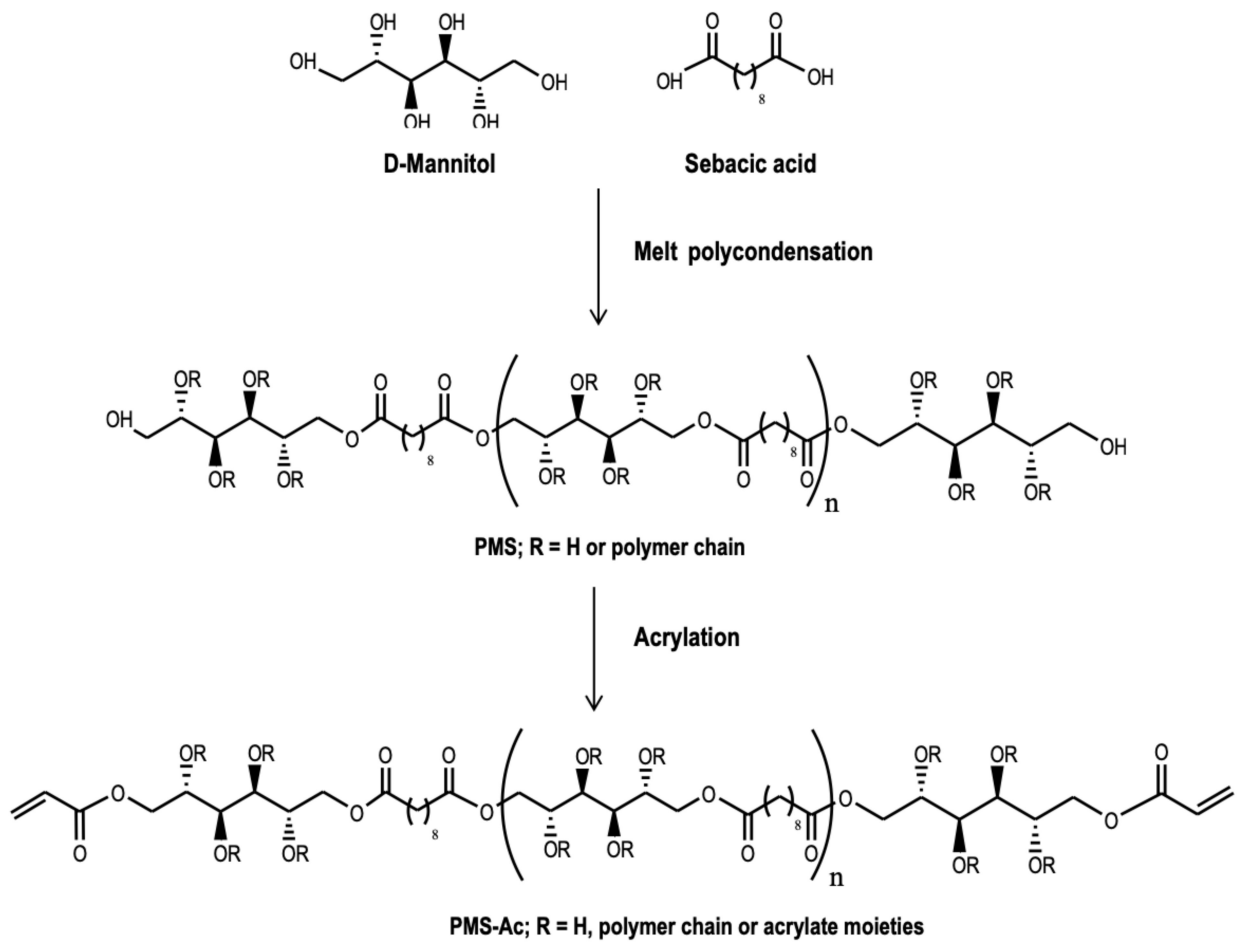
Synthesis of the PMS and its functionalization to synthesize PMSAc.

The obtained polymer was dissolved at 50 mg/mL (5% *w*/*v*) in anhydrous DMF under nitrogen flow in a three-neck round-bottomed flask. The solution was cooled to 0 °C and the acrylation was performed by slowly dropping molar equivalents (768 mM) of TEA and acryloyl chloride into the reaction. The mixture was stirred for 24 h at room temperature. Afterwards, ethyl acetate was added to the reaction and vacuum-filtered to remove the TEA salt. The process was repeated until a clear solution was obtained. The ethyl acetate was removed by rotary evaporation, and the resulting viscous mixture was precipitated into a hydrochloric acid solution (32 mM). Afterwards, the aqueous medium was decanted. The solid was subsequently washed with HCl solution and dried with magnesium sulphate. The obtained functionalized PMS (PMSAc) was protected from light and stored in the cold to await further use (see Scheme 1).

### 2.3. Fiber Preparation

The electrospinning solutions were prepared from a mixture of PLA and PMS, or PMSAc, using a ratio of 90/10 wt%, and dissolved in CHCl_3_/DMF 90:10 (*v*/*v*). The solutions were subjected to magnetic stirring for 24 h at room temperature to achieve complete homogenization.

The electrospun polymeric fibers were prepared using a homemade electrospinner, equipped with a syringe needle connected to a high-voltage power supply with a horizontal configuration. The prepared solutions were transferred into a syringe and loaded into a pump, placed parallel to the ground and programmed to deliver the solution at a constant flow. To promote the collection of the fibers, a rotating drum was used. The employed electrospinning parameters were 16 kV, with a needle-to-collector distance of 12 cm, a feeding rate of 1 mL/h, and rotor velocity of 1300 rpm. The electrospinning process was carried out at room temperature, and the relative humidity was around 30%. The obtained non-woven mats were vacuum-dried at room temperature for 24 h to remove any possible solvent residues. The electrospun fibers formed from pure PLA were also prepared for comparison with those loaded with PMS or PMSAc.

### 2.4. Surface Functionalization of the Fibers

Thiol-ene chemistry was employed to functionalize the electrospun fibers loaded with PMSAc and demonstrate the accessibility of the acrylate groups at the surface. The fiber mats (1 × 1 cm^2^) were incubated in 4 mL of a methanol solution containing 7-mercapto-4-methylcoumarin (0.004 mmol) and trimethylamine (0.016 mmol) at room temperature. After 72 h, the samples were washed with methanol several times to remove any residual reagents and dried.

### 2.5. Characterization Techniques

The number-average molecular weight (M_n_) and dispersity (Đ) of the PMS were determined by size-exclusion chromatography (SEC), using a chromatographic system (Waters Division Millipore) equipped with a Waters model 410 refractive-index detector, using N,N-dimethylformamide (Scharlau, 99.9%) containing 0.1% of LiBr as the eluent at a flow rate of 1 mL/min at 50 °C. Poly(methyl methacrylate) standards (Polymer Laboratories Ltd., Church Stretton, UK) were used to calibrate the system. The PMS and PMSAc were characterized by proton and carbon nuclear magnetic resonance spectroscopy (^1^H- and ^13^C- NMR), using a TM Bruker DPX 400 (400 MHz) spectrometer, at room temperature (128 scans, 1 s relaxation delay). Fourier transform infrared spectroscopy (FTIR) spectra were recorded on a Bruker Alpha instrument with a universal attenuated total reflectance (ATR) sampling accessory module. The theoretical depth penetration of the ATR–FTIR was 2.01 µm (Diamond crystal, refractive index: 2.4 and angle of incidence: 45°) and the spectral resolution was 0.4 cm^−1^. The electrospun fibers were characterized by scanning electron microscopy (SEM) using a Philips XL30 microscope. The orientation and diameter size were determined from the SEM images using Image J software. The thermal behavior of the electrospun fibers was analyzed by differential scanning calorimetry (DSC) using a TA Q2000 instrument (TA Instruments, New Castle, DE, USA) with dry nitrogen (50 cm^3^/min). The samples were equilibrated at −60 °C and heated to 200 °C at 10 °C/min. The glass transition temperatures (T_g_), cold crystallization temperatures (T_cc_), melting temperatures (T_m_), and the melting enthalpy (ΔH_m_) were obtained from the first heating scan. The crystallinity values were obtained by subtracting the enthalpy of the crystallization (∆H_c_) from the melting enthalpy (∆H_m_) and dividing it by the melting enthalpy, corresponding to a single PLA crystal (∆H_m_^0^, perfect crystal = 93.1 J/g) [27]. Errors in the temperature determination, enthalpy calculation, and the crystallinity were estimated at ±0.5 °C, ±4 J/g, and ±0.04 units, respectively.

The crystalline character of the fibers was analyzed by wide-angle X-ray diffraction (WAXS), employing synchrotron radiation at the beamline BL11-NCD-SWEET at ALBA (Cerdanyola del Vallés, Barcelona, Spain), at a fixed wavelength of 0.1 nm. A Rayonix detector was used for the WAXS (at about 14.6 cm from the sample, with a tilt angle of around 29 degrees) experiments. The spacing calibration was determined using silver behenate and Cr_2_O_3_ standards. The initial 2D X-ray images were converted into 1D diffractograms as a function of the inverse scattering vector, s = 1/d = 2 sin θ/λ, using the pyFAI python code (ESRF) that had been modified by the ALBA beamline staff. The mechanical properties were analyzed using a tensile (stretching) stage from Linkam (model TST350). At least ten rectangular samples were tested for each measurement. Errors in the determination of the Young modulus (E) and the elongation at break (ε) were estimated as the standard deviation.

The surface functionalization of the fibers with coumarin was studied by UV/vis absorption and fluorescence spectra, recorded using PerkinElmer Lambda-35 and PerkinElmer LS5OB spectrophotometers, respectively. The surfaces of the fiber mats were also observed under a fluorescence microscope (Nikon Eclipse TE2000-5), combined with a digital Nikon DS-Ri2 camera.

## 3. Results and Discussion

**Figure 1:**
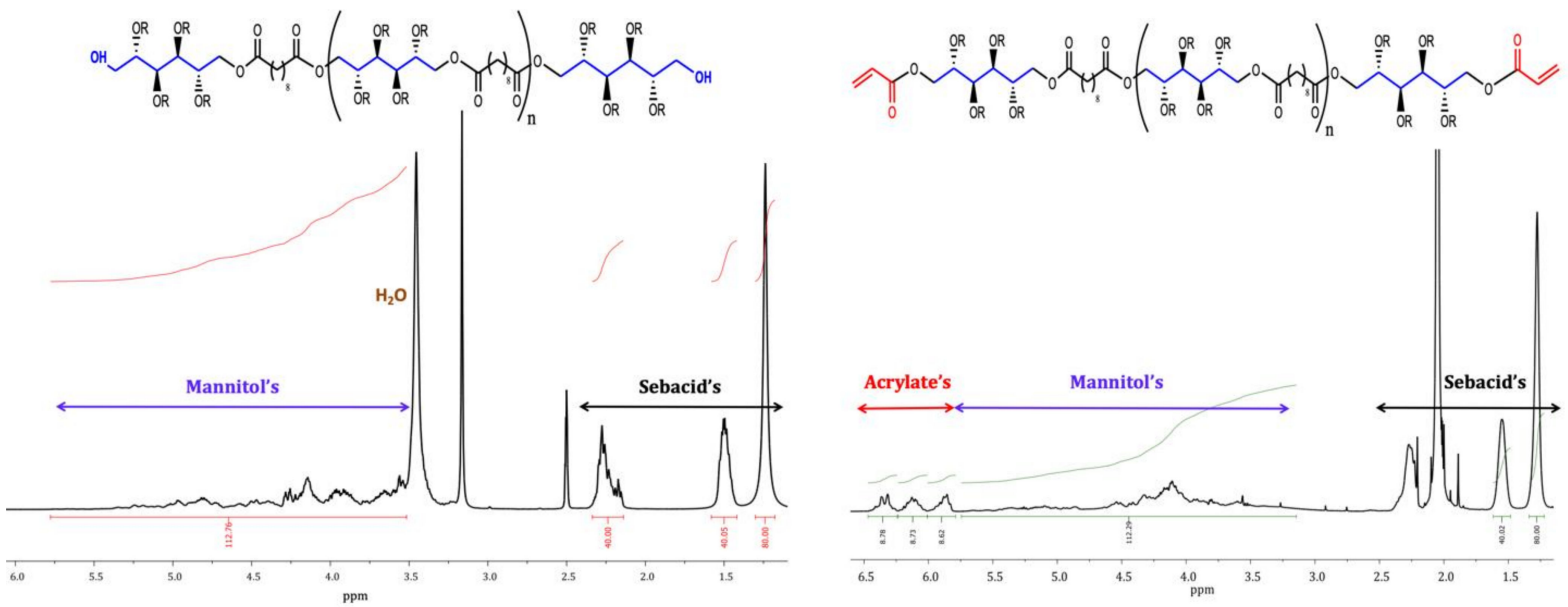
^1^ H-NMR spectra of the synthesized PMS and PMSAc.

The synthesis of the PMS from sebacic acid and D-mannitol in bulk was successfully performed, leading to a polymer with a low molecular weight. The M_n_ and Đ obtained by SEC were found to be 2480 g/mol and 1.22, respectively. This low molecular weight is, in principle, ideal for use as a polymer additive. The chemical composition of the PMS polymer was determined via ^1^H-NMR, by calculating the ratio of the sebacic acid to mannitol signal integrals and obtaining an experimental SA/MA composition of nearly the initial stoichiometric value. Peaks from the mannitol appeared at 3.5–5.5 ppm due to the central and terminal methylene units, while protons from the methylene units of the sebacic acid appeared at 1.3, 1.6, and 2.3 ppm (see Figure 1). The PMS polymer was subsequently modified by incorporating the acrylate groups into its structures through the hydroxyl groups. The acrylation degree was obtained by comparing the signal intensity of the methylene groups on the sebacic acid backbone (1.3 ppm) with the signal intensity of the acrylate groups (see Figure 1). The resulting value was found to be 87 ± 1% of the acrylation degree.

**Figure 2:**
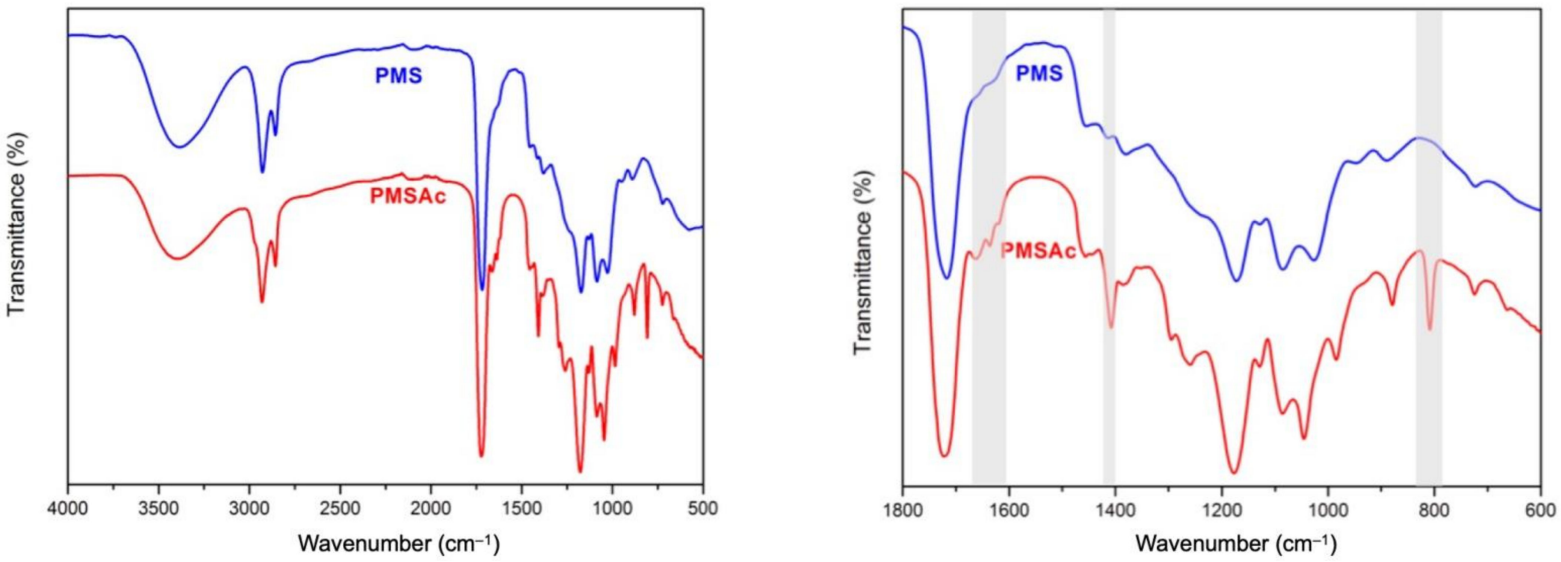
Right side: complete ATR–FTIR spectra of the PMS and PMSAc. Left side: magnified view of the 1800—600 cm^−1^ region.

Figure 2 shows the ATR–FTIR spectra of the PMS and PMSAc. Regarding the PMS spectra, the presence of the band at 1740 cm^−1^ also confirms the esterification reaction between the mannitol and sebacic acid. The characteristic broad peak around 3470 cm^−1^, associated with the hydroxyl stretch in the PMS, lowers its intensity in the PMSAc spectra after the acrylation reaction, which also confirms the modification of some hydroxyl moieties from the mannitol backbone. In addition to this, the bands at 2924 cm^−1^ and 2851 cm^−1^ observed in both the PMS and PMSAc correspond to methyl and alkane groups, while the ones shown at 1291—1050 cm^−1^ are associated with the stretching of the carboxyl bonds. Peaks associated with the acrylate group are seen at 808 cm^−1^ (C=CH_2_ twisting), 1407 cm^−1^ (C=CH_2_ scissoring), and 1636 cm^−1^ (C=CH_2_ stretching). These peaks are absent in the PMS polymer.

**Figure 3:**
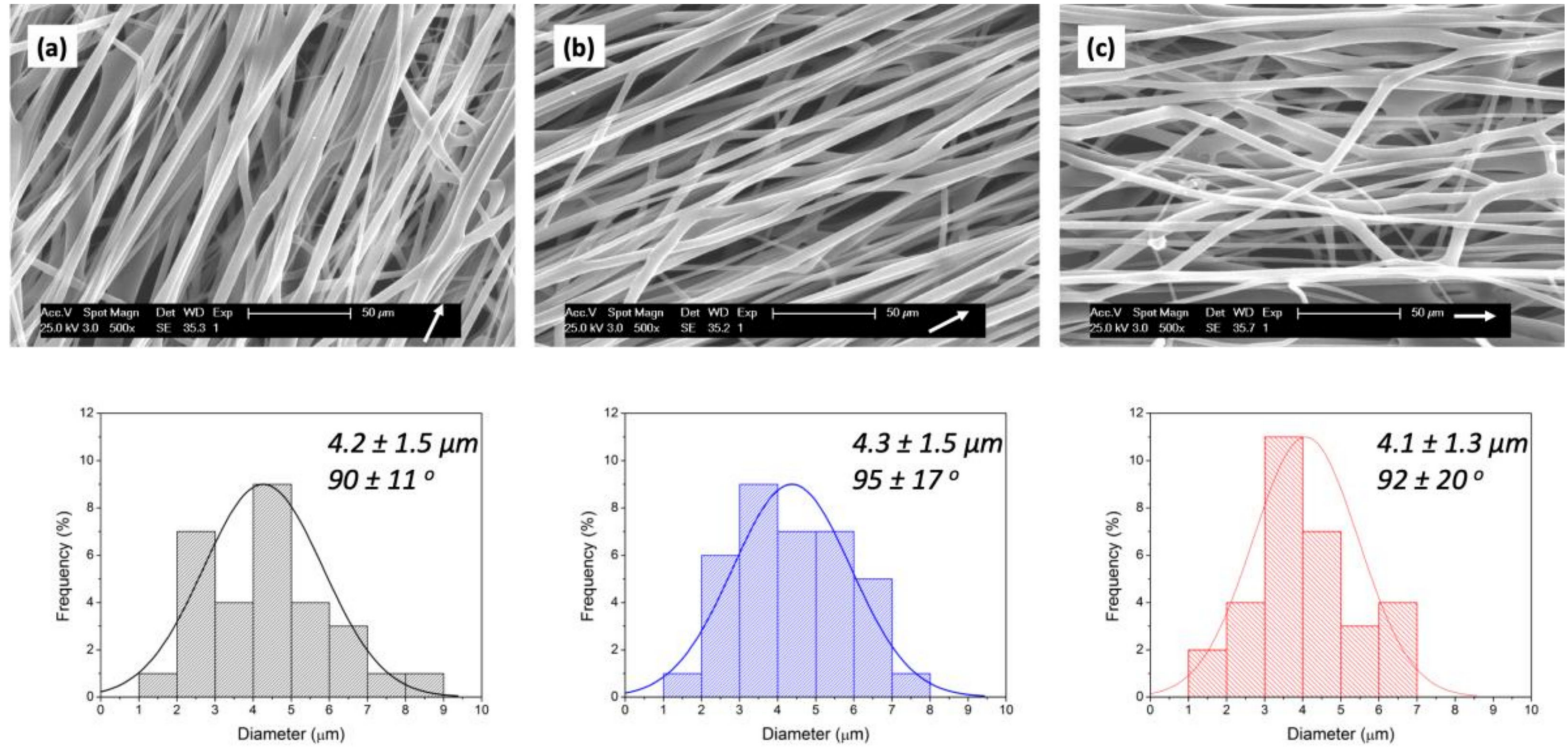
SEM images of the electrospun fibers and the corresponding size histograms and orientations of (**a**) PLA, (**b**) PLA/PMS, and (**c**) PLA/PMSAc. (White arrows indicate the orientation of the rotatory collector).

Next, both synthesized polymers, the PMS and PMSAc, were incorporated into PLA electrospinning solutions for the preparation of the polymer blend fibers. SEM images of the electrospun fibers of the neat PLA and the blends with PMS and PMSAc are displayed in Figure 3. As observed from the figure, the size of the fibers is maintained with the incorporation of the PMS and PMSAc, with an average value of 3 μm. It is worth mentioning that the rotatory collector clearly induced the alignment of the PLA fibers in the rotatory collector direction. When the PMS and PMSAc were incorporated into the PLA, the alignment was slightly altered, progressing from 90° for the PLA to 95, and 92° for the fibers loaded with PMS and PMSAc, respectively, as observed from Figure 3. However, there was still a significant alignment in the PLA–PMS based fibers. This alignment of the nanofibers contributes towards the promotion of their applicability in the biomedical field. In particular, aligned nanofibers are potential candidates for use in wound healing applications, since they mimic the extracellular matrix [3,28]. Moreover, it has been demonstrated that an increased alignment of the nanofibers can improve cell viability as compared to the random fibers [12].

In the case of the PLA fibers, the uniaxial alignment also impacted the mechanical properties, which improved significantly compared to the random ones. Additionally, the alignment can also promote the development of crystalline structures (α’-, β-) [29], as will be further described.

**Figure 4:**
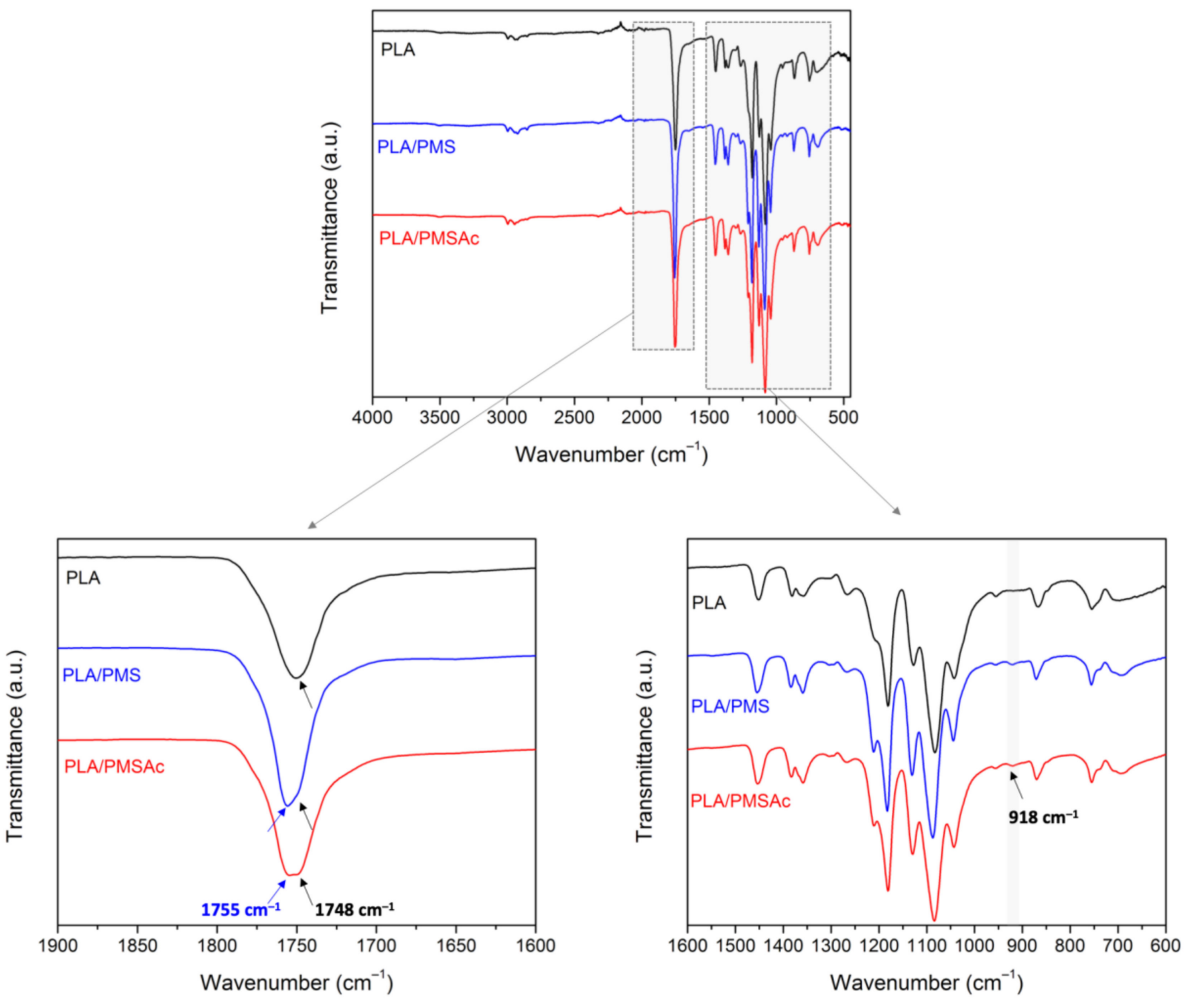
ATR–FTIR spectra of the electrospun fibers in the region of 1800–600 cm^−1^.

In order to confirm the compositions of the electrospun fibers, in Figure 4, the ATR–FTIR spectra of the PLA, PLA/PMS, and PLA/PMSAc electrospun fibers are shown. In the spectrum corresponding to the PLA fibers, the stretching vibration of the C=O group is observed at 1750 cm^−1^. The bands at 1450 and 1360 cm^−1^ are attributed to the symmetric and asymmetric bending of the methyl groups, and the band at 1380 cm^−1^ to the –CH deformation. At 1185 and 1085 cm^−1^, the symmetric ester -C-O stretching band and -C-O-C asymmetric stretching band can be observed, respectively.

The introduction of the PMSs slightly modified the PLA spectrum. For instance, changes in the stretching vibration of the C=O group were observed, showing the presence of a new band at 1755 cm^−1^, associated with the incorporation of the PMS and PMSAc (Figure 2). Additionally, a new band appeared at 918 cm^−1^, as highlighted in the figure, which can be associated with the development of a crystalline structure, in particular an α-form, due to the incorporation of the PMS and PMSAc. This result was also confirmed by XRD, and it is further explained below.

**Figure 5:**
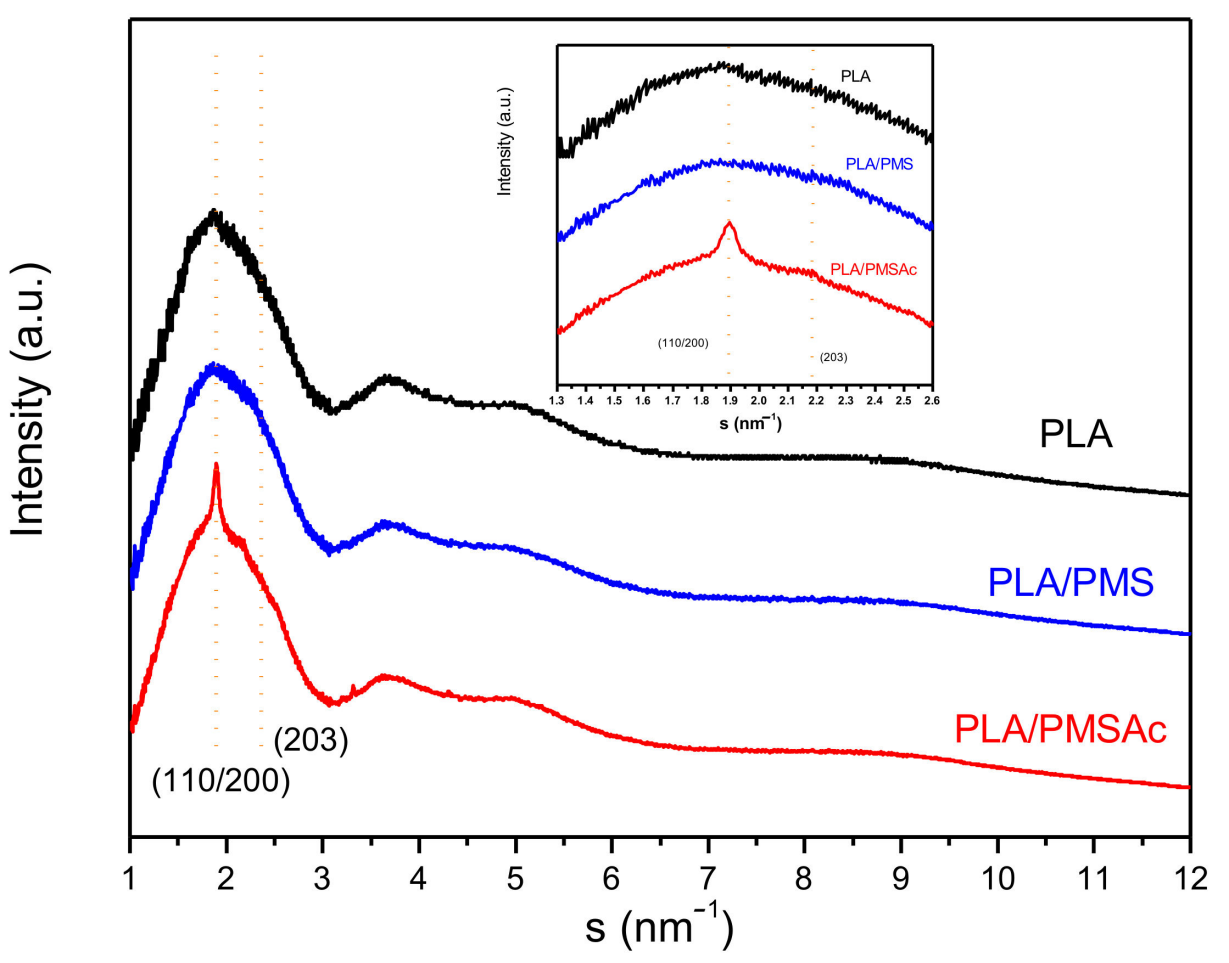
Wide-angle X-ray diffraction patterns of the PLA, PLA/PMS, and PLA/PMSAc fibers.

The PLA exhibited polymorphism, with four different crystal forms (named α-, β-, γ-, and ε-forms) and two disordered modifications of the α-form (named α′ and α″). It is known that the physical properties of polymers are strongly affected by crystal polymorphism. Therefore, the analysis of obtained PLA fibers is essential for understanding their behavior. Figure 5 displays the corresponding WAXS profiles of the electrospun fibers at room temperature. The use of synchrotron radiation allowed us to detect small peaks that would be imperceptible with CuKα radiation. The PLA fibers pattern revealed its amorphous character, presenting with very low, almost imperceptible crystallinity. This was also the case for the PLA/PMS blend fibers. However, in the blend of fibers with PMSAc, the crystallinity began to appear with visible reflections.

The α form of the PLA, a pseudo-orthorhombic structure with each unit cell containing two 10_3_ helical chains, presented a similar chain conformation to the α′ form, but, in this case, it was more disordered. In the pattern, we can distinguish a clear reflection at 1.86 nm^−1^ (110/200) and diffuse (203) at 2.12 nm^−1^, which can be attributed to both forms. The α-form is obtained by isothermal crystallization above 120 °C or by stretching a solution-spun PLA fiber at relatively low drawing temperatures and/or low hot-draw ratios [30]. The α′-form crystals are developed by melt or cold crystallization at lower temperatures (below 100 °C), and a mixture of α- and α’-form crystals is formed in the intermediate temperature region between 100 and 120 °C [31,32]. The diffraction profiles of α and α′ forms are quite different at *q* values of >15 nm^−1^ [33,34]. In the present work, the crystalline fractions did not allow us to distinguish between forms.

**Figure 6:**
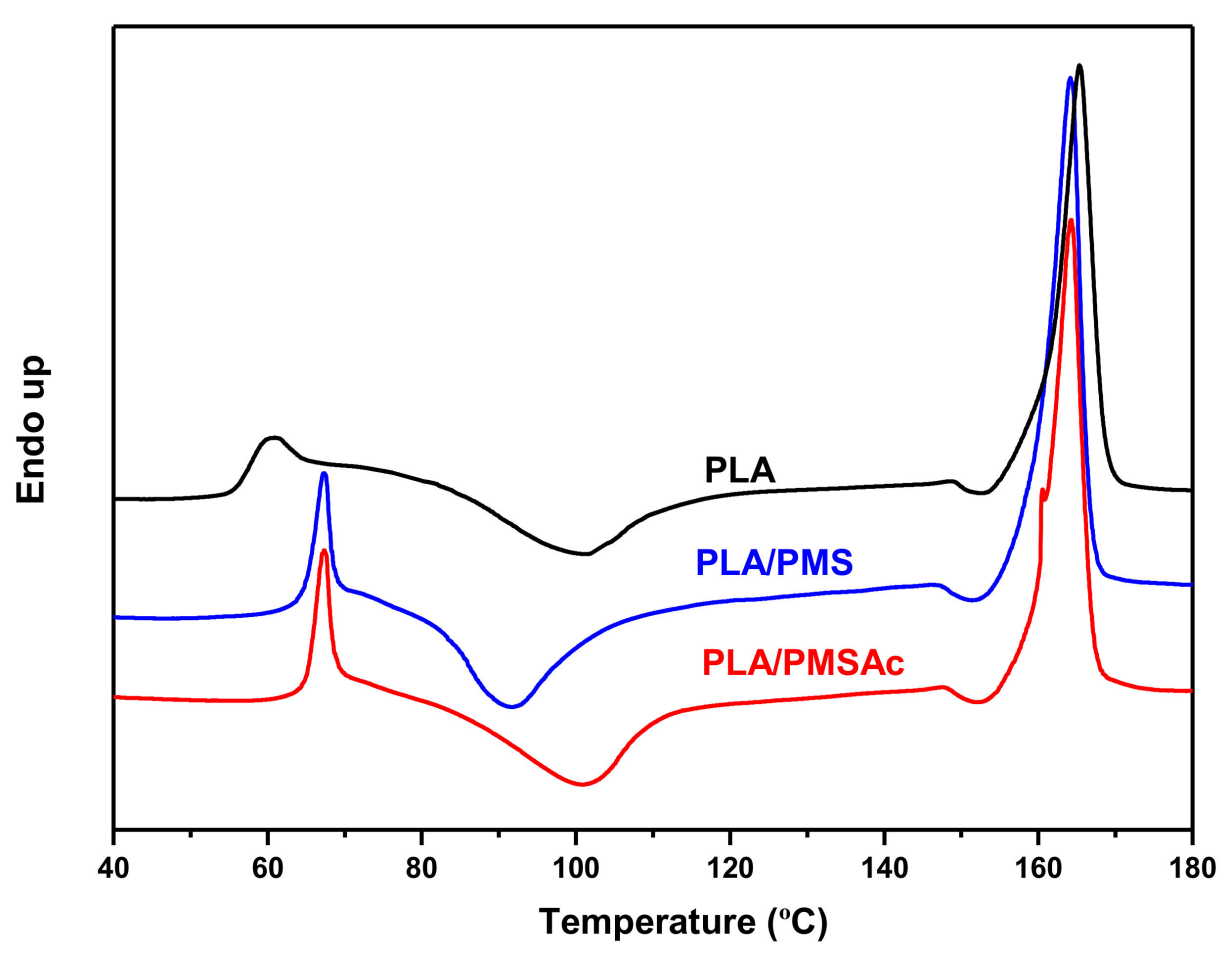
DSC curves of the different electrospun fibers.

In the following stage, the thermal analysis of the samples was performed to determine the corresponding thermal transitions in the electrospun fibers. In Figure 6, we can observe the glass transition temperature (T_g_) of the PLA, which presented with physical aging. Subsequently, the cold crystallization process began, followed by the melting of the crystalline units. It is clear that some recrystallization phenomena occur as soon as PLA begins to melt, and that this melting begins just after the cold crystallization peak [34,35]. The D-lactic acid percentage of the PLA chain influences the melting process and the ratio between the α and α’ forms, which correlated with the current case, with 2% of D-lactide in the PLA.

Our analysis revealed similar characteristic parameters as those reported in the literature [35,36,37,38]. The incorporation of the PMS and PMSAc in the fibers slightly changed this behavior. The T_g_ values of the blends were higher than that of the neat PLA (see Table 1). This fact could be attributed to the inter- and/or intramolecular hydrogen bonding between the PLA and PMSs. The PLA/PMS fibers also began their cold crystallization at a lower temperature, as did the melting. However, although the T_m_ was similar, the T_cc_ increased up to the PLA values when the acrylated PMS was incorporated into the blend. Moreover, the estimation of the crystallinity degree by DSC clearly confirms that observed in the WAXS measurements. In addition to this, the presence of the endothermic peak close to 155 °C present in each sample is also indicative of the presence of α’ form [34].

**Figure 7:**
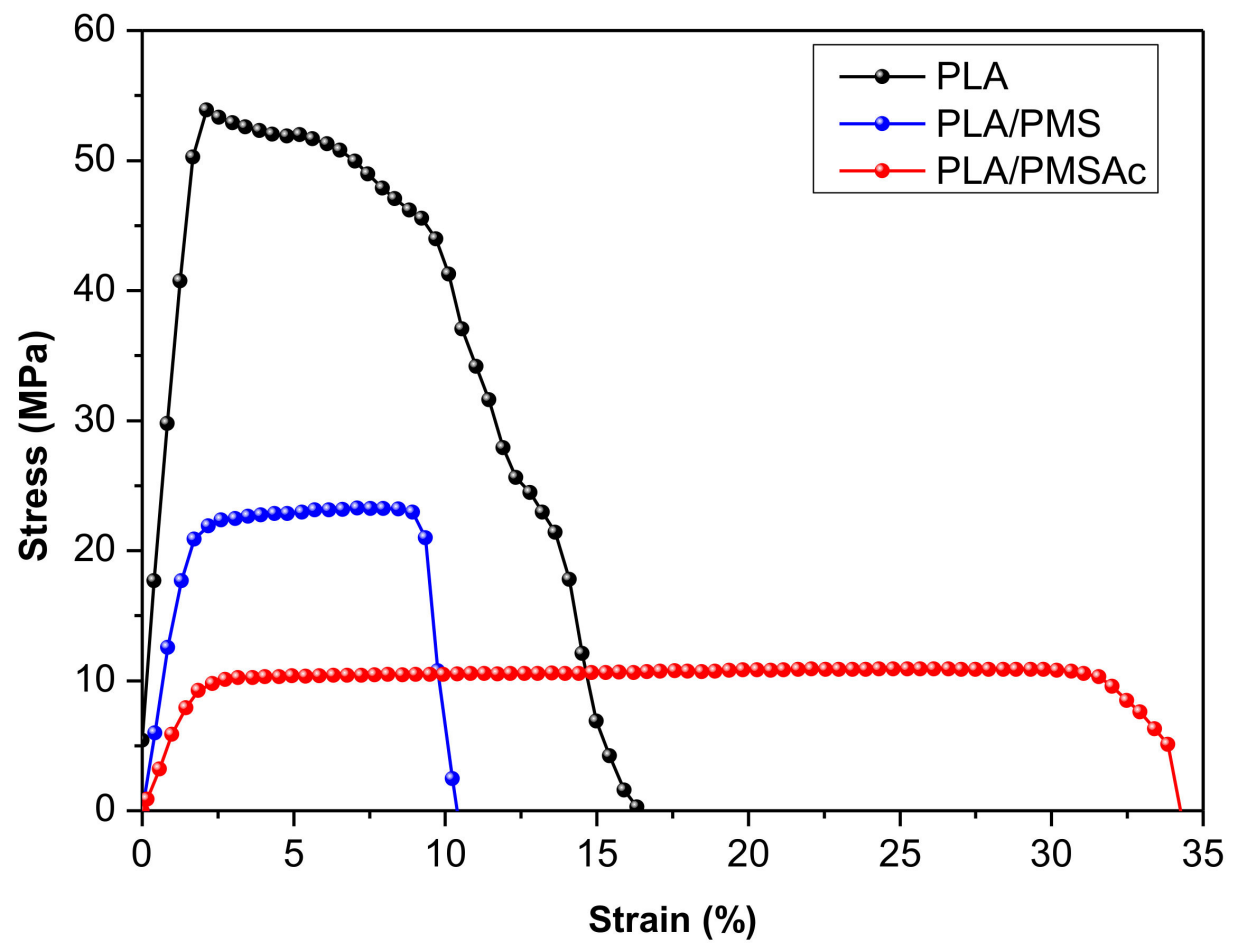
Stress–stain curves of the electrospun fibers of PLA, PLA/PMS, and PLA/PMSAc at 10 mm/min.

Next, the mechanical properties of all the obtained fibers were analyzed by uniaxial tensile experiments at room temperature, tested at a 10 mm/min stretching rate. Figure 7 depicts the representative profiles of the stress–strain curves of the obtained fibers, and Table 2 collects the corresponding values of the Young modulus and elongation at break. It is worthy noting that the attained E values were attributed to the fiber alignment, as mentioned before. As can be clearly observed, the incorporation of only 10 wt% of PMS or PMSAc into PLA fibers made the system more ductile, due to the elastomeric features of the PMS. The elongation at break (ε) increased, whereas the tensile strength (σ_max_) and the Young modulus (E) decreased, in comparison with the PLA fibers. In PLA fibers, typically a necking is produced before the fracture; thus, the incorporation of the PMSs prevented this and the strength was maintained before fracture. The fibers with the unmodified PMS almost duplicated in their strain capacity, and, in the case of PMSAc, this behavior was even more pronounced. The high values of the elongation at break in the fibers loaded with the PMSAc could be attributed to modifications of the hydrogen bonding (intra and intermolecular) interactions due to the acrylation reaction.

**Figure 8:**
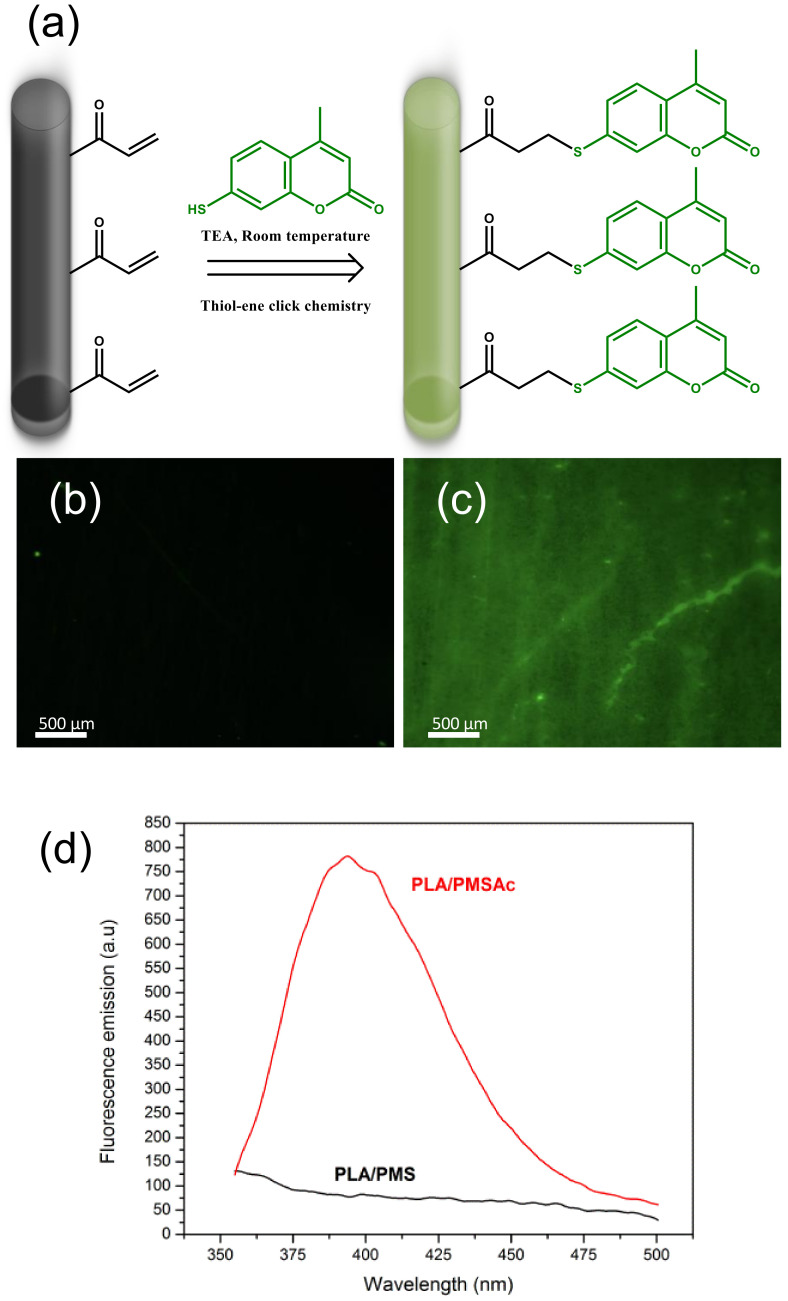
(**a**) Schematic illustration of the preparation process of the modification of the fiber surfaces with thiol-ene click chemistry. (**b**,**c**) Fluorescence micrographs of the PLA/PMS and PLA/PMSAc fibers, respectively, after thiol-ene reaction with mercapto-coumarin over the same exposure time. (**d**) Fluorescence spectra of the PLA/PMS and PLA/PMSAc fibers, respectively, after thiol-ene reaction with mercapto-coumarin using an excitation wavelength of 325 nm.

Therefore, the incorporation of a low percentage of this PMSAc polymer appears to be a promising alternative method for improving the ability of the material to bend and deform. Furthermore, PMSAc has reactive acrylate groups, which also confer surface activity to the fibers, and shows the potential for further chemical modification on the surface, such as grafting, crosslinking, or thiol-ene click chemistry reactions. The thiol-ene Michael addition of a thiol to an activated vinyl group, such as acrylate, offers a fast, highly efficient, and metal catalyst-free conjugational approach to the covalent attachment of functional compounds to surfaces in mild conditions [39,40,41]. To study the accessibility of the acrylate functional groups at the surface of the fibers, a Michael addition of the activated acrylate groups of PMSAc at the fiber surface with a mercapto-coumarin, used as a fluorescent probe, was carried out in the presence of triethylamine as a catalyst at an ambient temperature (Figure 8a). The surface reaction was successfully performed in mild conditions and tested by fluorescence microscopy and spectroscopy (Figure 8b–d).

As shown in Figure 8, the PLA/PMSAc fibers containing available acrylate groups showed strong fluorescence after the addition of the thiol functional dye, whereas the PLA/PMS fibers used as a control showed no fluorescence. Therefore, this confirmed the successful modification of the PLA/PMSAc fiber surfaces.

## 4. Conclusions

In summary, this work described the synthesis of biobased PMS and vinyl functionalized PMS, which were used as components of PLA-based electrospun fibers for the purpose of enhancing their mechanical properties. Partially aligned fibers containing 90% of PLA and 10% of the resulting low-molecular weight PMS or PMSAc were successfully obtained by electrospinning. These fibers presented with a low degree of crystallinity, as demonstrated by WAXS and DSC measurements, although the incorporation of the PMS and PMSAc slightly increased the crystallinity and augmented the T_g_ of the samples. Remarkably, the elasticity of the PLA-based fibers was enhanced through the addition of low levels of PMS or PMSAc, while the attained maximum strength was retained without a sudden drop before fracture, in contrast with what occurred in the neat PLA fibers. These phenomena occurred more markedly in the PLA/PMSAc fibers and, in addition, the acrylated polyester also demonstrated the possibility for further functionalization through the vinyl groups, which was demonstrated by the attachment of a thiol functional dye via Michael addition to the surface of the PLA-based fibers. Therefore, the incorporation of PMSAc at a low level to the PLA-based fibers enhanced their elasticity and toughness, and also provided functional groups at the surface that were available for further chemical modifications, such as grafting, crosslinking, or bio-conjugation through chemical reactions.

## Figures and Tables

**Table 1:** Thermal parameters obtained from the different fibers.

Fibers	T_g_ (°C)	T_cc_ (°C)	ΔH_cc_ (J/g)	T_m_ (°C)	ΔH_m_ (J/g)	X_c_ (%)
PLA	60	101	32	165	37	5
PLA/PMS	67	92	33	164	39	6
PLA/PMSAc	67	101	33	164	41	9

Errors in the temperature determination, enthalpy calculation, and the crystallinity were estimated at ±0.5 °C, ±4 J/g, and ±0.04 units, respectively.

**Table 2:** Mechanical parameters obtained from the different fibers.

Fibers	E (GPa)	σ_max_ (MPa)	ε (%)
PLA	2.2 ± 0.4	38 ± 8	8 ± 3
PLA/PMS	1.2 ± 0.2	31 ± 3	14 ± 2
PLA/PMSAc	0.8 ± 0.1	13 ± 4	31 ± 6

## Data Availability

Data sharing not applicable.
